# A Surge in Malaria Cases in the Eastern Health Region of Saudi Arabia During the COVID-19 Pandemic

**DOI:** 10.7759/cureus.37740

**Published:** 2023-04-17

**Authors:** Mousa J Alhaddad, Ali Alsaeed, Ridha H Alkhalifah, Makarem A Alkhalaf, Mohammed Y Altriki, Arif A Almousa, Mohammed J Alqassim, Fatimah Alibrahim

**Affiliations:** 1 Department of Internal Medicine, Dammam Medical Complex, Dammam, SAU; 2 Department of Internal Medicine, Infectious Disease Division, Dammam Medical Complex, Dammam, SAU; 3 College of Clinical Pharmacy, Imam Abdulrahman Bin Faisal University, Dammam, SAU

**Keywords:** saudi arabia, sars-cov-2, covid-19, rapid detection test, primaquine, plasmodium vivax, plasmodium falciparum, malaria

## Abstract

Background

Malaria transmission was stopped on most of the vast area of the Kingdom of Saudi Arabia. However, the pandemic of coronavirus disease (COVID-19) has negatively affected the efforts to control malaria. For instance, COVID-19 was reported to induce a relapse of malaria that is caused by *Plasmodium vivax*. Furthermore, physicians’ attention toward COVID-19 can only result in neglect and delayed diagnosis of complicated malaria cases. These factors, among others, might have contributed to an increase of malaria cases in Dammam, Saudi Arabia. Thus, this study was conducted to examine the effects of COVID-19 on malarial cases.

Methods

The medical records of all patients who were treated at Dammam Medical Complex for malaria between July 1, 2018, and June 30, 2022, were reviewed. Malaria cases were compared between the pre-COVID-19 period (between July 1, 2018, and June 30, 2020) and the COVID-19 period (between July 1, 2020, and June 30, 2022).

Results

A total of 92 malaria cases occurred in the total study period. There were 60 cases of malaria in the COVID-19 period as opposed to only 32 cases in the pre-COVID-19 period. All the cases were imported from the endemic Saudi southern areas or from outside the country. Eighty-two patients (89.1%) were males. Most of them were Sundaneses (39 patients, 42.4%), Saudis (21 patients, 22.8%), and tribal peoples (14 patients, 15.2%). Fifty-four patients (58.7%) were infected with *Plasmodium falciparum*. Seventeen patients (18.5%) were infected with *Plasmodium vivax*. Another 17 patients (18.5%) had a mixed infection with both *Plasmodium falciparum* and *Plasmodium vivax*. A trend toward more infected stateless tribal patients was observed in the COVID-19 period compared to the pre-COVID-19 period (21.7% vs 3.1%). A similar trend was noticed for mixed malarial infections with both *Plasmodium falciparum* and *Plasmodium vivax* (29.8% vs 0%) with a P value of less than 0.01.

Conclusion

Malaria cases were almost doubled during the COVID-19 pandemic as compared to the pre-pandemic era signifying the negative effects of the pandemic on malaria epidemiology. The cases increased for a variety of causes that include alternation of health-seeking behaviors, changes in healthcare structures and regulations, and the interruption of malaria preventive services. Future research is needed to study the long-term effects of the changes imposed by the COVID-19 pandemic and to mitigate the effects of any future pandemic on malaria control.

As two patients from our cohort were diagnosed with malaria based on blood smears, although they had negative rapid detection tests (RDTs), we recommend testing all the patients who are suspected to have malaria with both RDTs and peripheral blood smears.

## Introduction

Globally, 229 million malaria cases and 409 thousand deaths from malaria occurred in 2019 [1]. In Saudi Arabia, malaria transmission was stopped on the majority of the vast area of the country, but it continues to be endemic in its southwestern region in Tohama coastal area, Jazan Region, and the Saudi Arabia-Yemen border [2-4] with the continuing conflict in Yemen as a contributing obstacle for the elimination of malaria in Saudi Arabia [5].

The pandemic of coronavirus disease (COVID-19) has negatively affected the efforts to control malaria as it has caused a shortage of antimalarial agents and disturbed malaria preventive services [1,6]. Moreover, travelers might not get adequate malaria prophylaxis for the fear of getting COVID-19 if they visited the hospitals during the pandemic [7]. Furthermore, physicians’ attention toward COVID-19 can only result in neglect and delayed diagnosis of complicated malaria cases [8].

Hence, this study was conducted to evaluate the effects of COVID-19 on malaria cases in Dammam, Saudi Arabia.

This article was previously presented as a poster presentation at the 17th Annual Conference of the Saudi Society of Medical Microbiology and Infectious Diseases on March 1, 2023.

## Materials and methods

The study was a retrospective study. All malaria cases were treated at the Dammam Medical Complex, the largest governmental secondary hospital in the Eastern Health Region, which extends over the central part of the Saudi Eastern Province (excluding Al-Ahsa and Hafr Al-Baten Health Regions) and covers Dammam and its neighboring cities, between July 1, 2018, and June 30, 2022. The cases were grouped into two groups. The first group included the patients who presented between July 1, 2018, and June 30, 2020 (the pre-COVID-19 period). The second group included the patients who presented between July 1, 2020, and June 30, 2022 (the COVID-19 period). Notably, the Saudi Ministry of Health reported the first case of COVID-19 in Saudi Arabia on March 2, 2020 [9]. However, the first few months that followed this date were included in the pre-COVID-19 period, as it was not likely that the COVID-19 pandemic resulted in immediate effects on malaria cases, especially since the number of COVID-19 cases in these months was still low and that the Saudi healthcare system was not greatly affected by the pandemic yet.

The two groups were compared in the number of malaria cases, the identified malaria species, and the patient's demographics, initial laboratory investigations, and complications.

The data were analyzed using the Python programming language version 3.7.6 (Python Software Foundation, Wilmington, Delaware, USA) with the use of the SciPy library 1.4.1 (Enthought, Inc., Austin, Texas, USA), and Statsmodels module (v0.11.1, Python package). Descriptive statistics (i.e., mean, standard deviation, count, and percentage) were calculated as necessary. Categorical variables were compared with the Chi-square test, and continuous variables were compared with the two-sample t-test. A p-value of less than 0.05 was assumed to indicate statistical significance.

The research project was approved and monitored by the Dammam Medical Complex Institutional Review Board (IM-03), and all data were used only for research purposes. 

## Results

**Figure 1:**
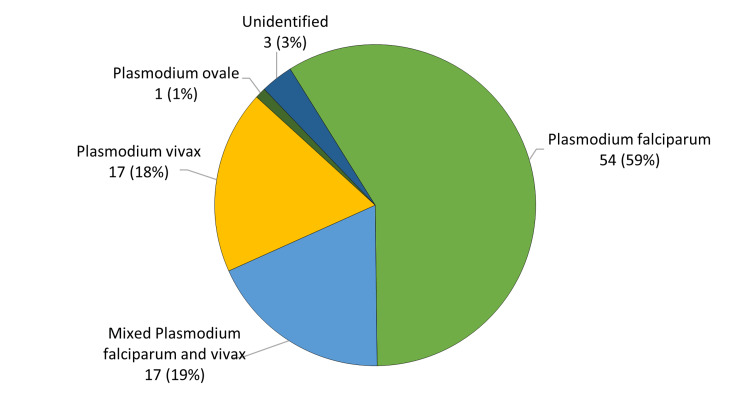
Malaria types (n = 92).

A total of 92 malaria cases were treated at the Dammam Medical Complex between July 1, 2018, and June 30, 2022. The patients consisted of 82 male and 10 female patients (89.1% and 10.9%, respectively), with a male-to-female ratio of 8.2. Most of the patients were Sundaneses (39 patients, 42.4%), Saudis (21 patients, 22.8%), or stateless tribal persons (14 patients, 15.2%). The mean ± standard deviation for the patients' age was 33.7 ± 11.1 years. Twenty patients (21.7%) had a personal previous history of malaria. Fever (92 patients, 100%) was the most common presenting symptom, followed by body pains (32 patients, 34.8%) and fatigue (19 patients, 20.7%). The mean ± standard deviation for the patients' duration of symptoms prior to presentation was 7.0 ± 7.2 days with a median of 5.0 days and a range from one day to 60 days. Eighty-one patients (88.0%) were admitted to the hospital for management. The remaining patients (11 patients, 12.0%) were managed in the emergency room. The mean ± standard deviation for the patients' parasitemia level was 2.2 ± 3.0% with a median of 1.0% and a range from 0.001% to 16.0%. Most of the patients were infected with *Plasmodium falciparum* alone (54 patients, 58.7%) followed by isolated *Plasmodium vivax* infection (17 patients, 18.5%) and mixed infection of both *Plasmodium falciparum* and *Plasmodium vivax* (17 patients, 18.5%). Only one patient (1.1%) was infected with *Plasmodium ovale*. The type of malaria parasite was not identified in three (3.3%) patients (Figure 1). 

**Figure 2:**
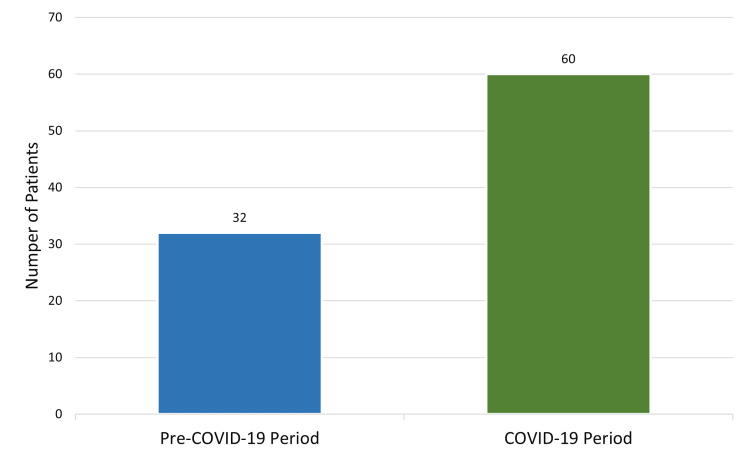
Malaria cases and periods of presentation (n = 92).

Thirty-two patients (34.8%) presented in the pre-COVID-19 period. The remaining 60 patients (65.2%) presented in the COVID-19 period (Figure 2).

The patient's demographics and clinical presentations are given in Table 1.

**Table 1:** Patients' demographics and clinical presentations (n = 92).

Characteristic		n (%)
Age (mean ± SD, years)		33.66 ± 11.11
Weight (mean ± SD, Kg)		70.57 ± 15.95
Gender	Male	82 (89.13%)
	Female	10 (10.87%)
Nationality	Sudanese	39 (42.39%)
	Saudi	21 (22.83%)
	Tribes	14 (15.22%)
	Yemeni	6 (6.52%)
	Others	12 (13.04%)
Diabetes mellitus		6 (6.52%)
Hypertension		4 (4.35%)
Personal past history of malaria		20 (21.74%)
Personal history of visiting endemic areas	Sudan	39 (42.39%)
	Saudi Arabia's southern region	30 (32.61%)
	Yemen	8 (8.7%)
	Oman	3 (3.26%)
	Other African countries	8 (8.7%)
	Other Asian countries	3 (3.26%)
	Unknown	1 (1.09%)
Duration of symptoms (mean ± SD, days)		7.02 ± 7.19
Symptoms	Fever	92 (100.0%)
	Body pains	32 (34.78%)
	Fatigue	19 (20.65%)
	Vomiting	39 (42.39%)
	Headache	29 (31.52%)
Parasitemia (mean ± SD, percentages)		2.23 ± 2.98
Malaria type	Plasmodium falciparum	54 (58.7%)
	Plasmodium vivax	17 (18.48%)
	*Plasmodium falciparum* and *P. vivax*	17 (18.48%)
	Plasmodium ovale	1 (1.09%)
	Unidentified	3 (3.26%)
Malaria screen	Positive	88 (95.65%)
	Negative	2 (2.17%)
	Not tested	2 (2.17%)
Glucose-6-phosphate dehydrogenase (G6PD) status	Normal	49 (53.26%)
	Deficient	1 (1.09%)
	Not tested	42 (45.65%)
Encounter	Admitted into the hospital	81 (88.04%)
	Managed in the emergency room	11 (11.96%)
Period	COVID-19 period	60 (65.22%)
	Pre-COVID-19 period	32 (34.78%)

Thrombocytopenia was the most common hematological abnormality at presentation occurring in 80 patients (87.0%) with a mean platelet count of 88.3 ± 59.0 × 10^9^/L. Thirty patients (32.6%) had a hemoglobin of less than 12 g/dl. Leukopenia and leukocytosis were found in 19 (20.7%) and 7 (7.6%) patients, respectively. The patients' initial total and direct bilirubin levels were 2.6 ± 2.9 and 1.4 ± 2.4 mg/dl, respectively. The patients' initial laboratory results are given in Table 2.

**Table 2:** Patients' initial laboratory results (n = 92).

Characteristic	Mean ± SD	Normal range
White blood cells (WBCs)	6.16 ± 2.8	4-10 × 10^9^/L
Neutrophils	4.23 ± 2.33	2-7.5 × 10^9^/L
Lymphocytes	1.18 ± 0.84	1.5-4 × 10^9^/L
Monocytes	0.56 ± 0.37	0.2-1 × 10^9^/L
Eosinophils	0.09 ± 0.13	0.04-0.4 × 10^9^/L
Basophils	0.04 ± 0.05	0-0.1 × 10^9^/L
Hemoglobin	12.34 ± 2.07	11.5-15.5 g/dl
Platelets	88.34 ± 59.03	150-450 × 10^9^/L
Creatinine	1.21 ± 1.31	0.5-0.9 mg/dl
Total bilirubin	2.56 ± 2.86	0-1 mg/dl
Direct bilirubin	1.35 ± 2.36	0-0.2 mg/dl
Glucose	122.95 ± 49.16	74-106 mg/dl

Eighty patients (87.0%) were treated with intravenous artesunate with a duration of 4.0 ± 1.9 days. Artesunate-sulfadoxine-pyrimethamine (17 patients, 18.5%) and chloroquine (nine patients, 9.8%) were the most commonly used oral antimalarial agents. Primaquine was given to 27 patients (71.43% out of 35 patients infected with *Plasmodium vivax* or *Plasmodium ovale*). The patients' antimalarial medications are given in Table 3.

**Table 3:** Patients' antimalarial medications (n = 92).

Medication		n (%)
Intravenous artesunate		80 (86.96%)
Oral antimalarial agents	None	61 (66.3%)
	Artesunate-sulfadoxine-pyrimethamine	17 (18.48%)
	Chloroquine	8 (8.7%)
	Quinine and doxycycline	2 (2.17%)
	Quinine	2 (2.17%)
	Doxycycline	1 (1.09%)
	Chloroquine and doxycycline	1 (1.09%)
Primaquine		27 (29.35%)

Twenty-two patients (23.9%) developed an acute kidney injury (AKI). Shock or sepsis occurred in seven patients (7.6%). Four patients (4.4%) had cerebral malaria with all of them having impaired consciousness and delirium and none of them developing seizures. One patient (1.1%) infected with *Plasmodium falciparum* developed myocarditis and heart failure [10]. A different patient (1.1%) infected with *Plasmodium vivax* presented with a splenic rupture. Both diabetic ketoacidosis and hypoglycemia occurred also in one patient (1.1%). The mean ± standard deviation for the duration from presentation to discharge for the hospitalized patients was 5.1 ± 4.1 days. Six patients (6.5%) were admitted to the intensive care unit (ICU) and stayed there for 2.6 ± 2.0 days. Five patients (5.4%) relapsed after discharge and were readmitted, with a mean duration from discharge to readmission of 43.4 ± 35.5 days. A single patient died (1.1%) in this cohort. This patient was infected with *Plasmodium falciparum*, his diagnosis got delayed after multiple emergency room visits, and his presentation included cerebral malaria (impaired consciousness, sleepiness, and vertigo) with severe hypotonic hyponatremia (117 mmol/L). The patients' complications and outcomes are given in Table 4.

**Table 4:** Patients' complications and outcomes (n = 92). *Splenomegaly rates might be under-reported as only a few patients were evaluated by an abdominal ultrasound study.

Characteristic		n (%)
Acute kidney injury		22 (23.91%)
Shock or sepsis		7 (7.61%)
Cerebral malaria	Impaired consciousness	4 (4.35%)
	Convulsions	0 (0.00%)
Splenomegaly*		4 (4.35%)
Splenic rupture		1 (1.09%)
Myocarditis		1 (1.09%)
Hypoglycemia		1 (1.09%)
Diabetic ketoacidosis		1 (1.09%)
Hospitalization		81 (88.04%)
Intensive care unit (ICU) admission		6 (6.52%)
Relapse/readmission		5 (5.43%)
Death		1 (1.09%)

**Figure 3:**
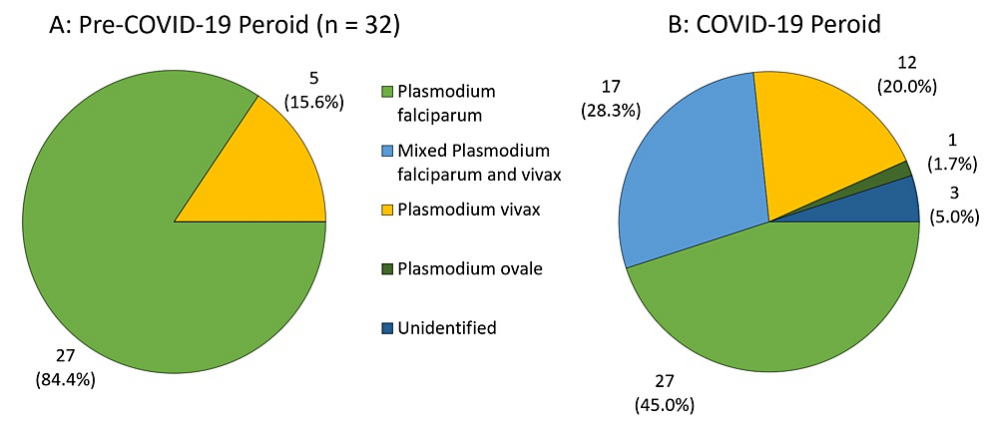
Types of malaria in the pre-COVID-19 and COVID-19 periods. Panel A: *Plasmodium falciparum *was the predominant cause of malaria in the pre-COVID-19 period. Panel B: *Plasmodium falciparum* remained the predominant cause of malaria in the COVID-19 period but cases of mixed *Plasmodium falciparum* and *Plasmodium vivax* started to occur.

A trend toward more infected stateless tribal patients was observed in the COVID-19 period compared to the pre-COVID-19 period (21.7% vs 3.1%). A similar trend was noticed for mixed malarial infections with both *Plasmodium falciparum* and *Plasmodium vivax* (29.8% vs 0%) with a P value of less than 0.01 (Figure 3).

There were no other significant differences between the two groups. Table 5 provides a detailed comparison between the patients in the pre-COVID-19 period and the patients in the COVID-19 period.

**Table 5:** Comparison between the malaria patients in the pre-COVID-19 period and the COVID-19 period. *Significant at a P value of less than 0.05.

Characteristic		COVID-19 period (n = 60)	Pre-COVID-19 period (n = 32)	P value
Age, mean ± SD, years		32.29 ± 9.53	36.23 ± 13.38	0.1059
Male sex, count (%)		52 (86.67%)	30 (93.75%)	0.4915
Nationality, count (%)	Sudanese	21 (35.0%)	18 (56.25%)	0.0777
	Saudi	13 (21.67%)	8 (25.0%)	
	Tribes	13 (21.67%)	1 (3.12%)	
	Yemeni	4 (6.67%)	2 (6.25%)	
	Other African	4 (6.67%)	3 (9.38%)	
	Other Asian	5 (8.33%)	0 (0.0%)	
Personal history of visiting endemic areas, count (%)	Saudi Arabia's Southern Region	22 (37.29%)	8 (25.0%)	0.4244
	Sudan	22 (37.29%)	17 (53.12%)	
	Yemen	6 (10.17%)	2 (6.25%)	
	Oman	2 (3.39%)	1 (3.12%)	
	Other African countries	4 (6.78%)	4 (12.5%)	
	Other Asian countries	3 (5.08%)	0 (0.0%)	
Diabetes mellitus, count (%)		4 (6.67%)	2 (6.25%)	0.7142
Hypertension, count (%)		3 (5.0%)	1 (3.12%)	0.9071
Personal history of malaria, count (%)		16 (26.67%)	4 (12.5%)	0.1923
Duration of symptoms, mean ± SD, days		6.32 ± 4.42	8.37 ± 10.6	0.2076
Parasitemia, mean ± SD, percentages		2.35 ± 3.1	1.93 ± 2.74	0.5865
Malaria type, count (%)	Plasmodium falciparum	27 (45%)	27 (84.38%)	0.0018*
	Plasmodium vivax	12 (20%)	5 (15.62%)	
	*Plasmodium falciparum* and *vivax*	17 (28.33%)	0 (0.0%)	
	Plasmodium ovale	1 (1.67%)	0 (0.0%)	
	Unidentified	3 (5.0%)	0 (0.0%)	
Initial white blood cells, mean ± SD, ×10^9^/L		6.14 ± 2.73	6.19 ± 2.98	0.9406
Initial hemoglobin, mean ± SD, g/dl		12.37 ± 2.22	12.28 ± 1.8	0.8423
Initial platelets, mean ± SD, ×10^9^/L		84.74 ± 58.45	95.19±60.47	0.4278
Initial creatinine, mean ± SD, mg/dl		1.26 ± 1.5	1.09 ± 0.84	0.5661
Initial direct bilirubin, mean ± SD, mg/dl		1.36 ± 2.29	1.32 ± 2.53	0.9435
Initial total bilirubin, mean ± SD, mg/dl		2.69 ± 2.86	2.3 ± 2.89	0.5479
Initial glucose, mean ± SD, mg/dl		129.76 ± 55.41	114.24 ± 39.16	0.2402
Use of intravenous artesunate, count (%)		55 (91.67%)	25 (78.12%)	0.1306
Duration of artesunate, mean ± SD, days		4.0 ± 1.98	4.0 ± 1.73	1
Use of primaquine, count (%)		21 (35.0%)	6 (18.75%)	0.1646
Acute kidney injury, count (%)		15 (25.0%)	7 (21.88%)	0.9378
Shock or sepsis, count (%)		6 (10.0%)	1 (3.12%)	0.4403
Impaired consciousness, count (%)		2 (3.33%)	2 (6.25%)	0.9071
Splenomegaly, count (%)		3 (5.0%)	1 (3.12%)	0.9071
Hospitalization, count (%)		56 (93.33%)	25 (78.12%)	0.0712
Duration of hospitalization, mean ± SD, days		5.34 ± 4.59	4.38 ± 2.43	0.3345
Intensive care unit admission, count (%)		3 (5.0%)	3 (9.38%)	0.7142
Death, count (%)		0 (0.0%)	1 (3.12%)	0.748

Malaria patients who developed an AKI were significantly older than the patients who did not develop any renal impairment (38.7 ± 12.2 vs 32.1 ± 10.4 years, P value <0.05). They contained more diabetic (18.2% vs 2.9%, P value <0.05) and hypertensive patients (18.2% vs 0%, P value <0.01). Additionally, they had higher levels of parasitemia (3.8 ± 4.4 vs 1.7 ± 2.2%, P value <0.01) and total bilirubin (3.9 ± 3.8 vs 2.1 ± 2.3 mg/dl, P value <0.01). Expectedly, they stayed longer in the hospital (7.9 ± 6.6 vs 4.1 ± 1.9 days, P value <0.001). The detailed comparison between the malaria patients based on developing AKI is shown in Table 6.

**Table 6:** Comparison between the malaria patients based on developing acute kidney injury (AKI). *Significant at a P value of less than 0.05.

Characteristic	No AKI (n = 70)	AKI (n = 22)	P value
Age, mean ± SD, years	32.08 ± 10.35	38.68 ± 12.18	0.0142*
Male sex, count (%)	62 (88.57%)	20 (90.91%)	0.932
Diabetes mellitus, count (%)	2 (2.86%)	4 (18.18%)	0.0409*
Hypertension, count (%)	0 (0.0%)	4 (18.18%)	0.0023*
Personal history of malaria, count (%)	15 (21.43%)	5 (22.73%)	0.867
Duration of symptoms, mean ± SD, days	6.62 ± 4.68	8.29 ± 12.19	0.3582
Parasitemia, mean ± SD, percentages	1.71 ± 2.19	3.83 ± 4.37	0.0098*
Initial white blood cells, mean ± SD, ×10^9^/L	5.83 ± 2.59	7.17 ± 3.22	0.052
Initial hemoglobin, mean ± SD, g/dl	12.34 ± 2.12	12.32 ± 1.96	0.9622
Initial platelets, mean ± SD, ×10^9^/L	95.19 ± 61.35	67.17 ± 46.23	0.0524
Initial creatinine, mean ± SD, mg/dl	0.89 ± 0.19	2.16 ± 2.41	0*
Initial direct bilirubin, mean ± SD, mg/dl	0.92 ± 1.8	2.64 ± 3.27	0.0025*
Initial total bilirubin, mean ± SD, mg/dl	2.1 ± 2.34	3.94 ± 3.76	0.0082*
Initial glucose, mean ± SD, mg/dl	122.68 ± 52.26	124.0 ± 37.1	0.9348
Use of intravenous artesunate, count (%)	60 (85.71%)	20 (90.91%)	0.7885
Duration of artesunate, mean ± SD, days	3.83 ± 1.69	4.5 ± 2.4	0.1748
Use of primaquine, count (%)	18 (25.71%)	9 (40.91%)	0.2727
Shock or sepsis, count (%)	5 (7.14%)	2 (9.09%)	0.8726
Impaired consciousness, count (%)	2 (2.86%)	2 (9.09%)	0.5148
Splenomegaly, count (%)	2 (2.86%)	2 (9.09%)	0.5148
Hospitalization, count (%)	60 (85.71%)	21 (95.45%)	0.3944
Duration of hospitalization, mean ± SD, days	4.05 ± 1.87	7.86 ± 6.64	0.0001*
Intensive care unit admission, count (%)	3 (4.29%)	3 (13.64%)	0.2917
Death, count (%)	1 (1.43%)	0 (0.0%)	0.5386

## Discussion

Malaria cases in Dammam Medical Complex have increased by 93% in the COVID-19 period compared to the pre-COVID-19 period. A similar surge of malaria cases was also noticed in other countries like Zimbabwe [11], Gabon [12], Bhutan [13], and Peru [14]. Likewise, there was a report of increasing cases of severe malaria following the COVID-19 pandemic among the French Armed Forces during their missions in the Sub-Saharan African countries [15]. Relatedly, 2021 was the highest year of imported malaria cases in Shanghai over the past 10 years [16].

Many factors might explain this surge in malaria cases, both locally in Saudi Arabia and globally following the COVID-19 pandemic. For instance, COVID-19 was reported to induce a relapse of malaria that is caused by *Plasmodium vivax* [17-19] and *Plasmodium ovale* [20]. Similarly, the Pfizer BioNTech (BNT162b2) COVID-19 vaccine, the first COVID-19 vaccine to be approved in Saudi Arabia [21] and the most used COVID-19 vaccine by Saudi citizens [22], was reported to cause recrudescence of subclinical chronic malarial infections [23]. Additionally, malaria patients may complain from fever, headache, and body pains, which are also reported commonly due to COVID-19. In consequence, the concerns of getting infected by the COVID-19 virus during the pandemic might have driven more of these patients to seek medical care and, hence, be diagnosed with malaria at the end [11].

The increase in malaria cases in the Dammam Medical Complex might also be related to the changes imposed on the healthcare system following the COVID-19 pandemic. Other centers in the Eastern Province were used to diagnose and treat some mild malaria patients with oral antimalarial agents. Following the pandemic, some of these centers were changed into COVID-19 centers and stopped managing non-COVID-19 patients. Others might have run out of hydroxychloroquine following the initial, false reports that it was effective against COVID-19 and might have been left with no other options to treat malaria. It is likely that the patients who would normally be treated at such centers were all referred to the Dammam Medical Complex, as it is the main malaria hospital in the region. Similarly, some private and specialized centers and hospitals tried to maintain a non-COVID-19 environment and referred all febrile patients who were suspected to have COVID-19 to Dammam Medical Complex, the primary COVID-19 hospital in the Eastern Province. Additionally, the royal order to treat all confirmed and suspected COVID-19 patients for free might have encouraged more patients who reside in Saudi Arabia illegally, a major vulnerable yet under-represented group [5], to seek medical care. It is likely also that COVID-19 caused restrictions to international travel, changing the usual travel destinations and leading to more local infections, as evident by the increasing number of patients who acquired malaria from visiting the endemic Saudi Arabia's southern areas during the COVID-19 period.

In Africa, the interruption of malaria services by the COVID-19 pandemic disturbed the mass distribution of long-lasting insecticide-treated nets (LLINs), and impacted many focal preventative measures such as seasonal malaria chemoprevention (SMC) and indoor residual spraying of insecticides (IRS) [1,13,24]. It also resulted in decreased access to effective antimalarial treatment [1]. It is expected that the preventive measures in Saudi Arabia have also been interrupted following the pandemic, though to a lesser degree.

One of the long-term effects of the COVID-19 pandemic on malaria control is the growing vaccine hesitancy that was associated with the introduction and distribution of COVID-19 vaccines. This hesitancy could hinder plans for developing and administering malaria vaccines [25]. Moreover, it is known that chloroquine sensitivity in *Plasmodium falciparum* could re-emerge with time, as occurred in the Jazan Region, where one-third of the isolates returned to be susceptible [26]. However, the wide use of chloroquine and hydroxychloroquine during the initial stages of the COVID-19 pandemic could delay their sensitivity re-emergence [6]. 

The deadliest type of malaria, *Plasmodium falciparum*, was the predominant malaria species in this study presenting either alone or mixed with *Plasmodium vivax* in 77% of the study patients. This matches the published data that found it to be the most common malaria type in Saudi Arabia [3,5]. *Plasmodium falciparum* predominated in both the pre-COVID-19 and the COVID-19 periods. However, rates of mixed infections increased during the COVID-19 period. All 17 cases of mixed *Plasmodium falciparum* and *Plasmodium vivax* infections in the study were in the COVID-19 period. This unmatched increase in mixed infections should be interpreted with cautions as the identification of the malaria species was not validated in a reference laboratory. 

Apart from the presumed effects of COVID-19 on malaria cases, the study revealed deficiencies in primaquine prescriptions for patients infected with *Plasmodium vivax* and *Plasmodium ovale*. About 29% of these patients were not given primaquine to eradicate hypnozoites after the initial antimalarial therapy resulting in five cases of relapse. A portion of these patients was managed without any involvement from infectious disease fellows and consultants signifying the importance of infectious disease consultation in managing malaria cases. Regardless, the main reason that primaquine was not given to many patients was that the glucose-6-phosphate dehydrogenase (G6PD) enzyme status was not readily available at the time of discharge. Fastening the release time of the G6PD results is expected to improve the rates of primaquine prescriptions.

It was also noted in this study that physicians should not rely too much on malaria rapid detection tests (RDTs) as they do not always detect malarial infections [27,28]. Two patients from our cohort were diagnosed with malaria based on blood smears, although they had negative RDTs. Both patients were from Sudan, a country among many that reported cases of RDT-negative malaria due to parasites carrying hrp2/3 deletions [29,30]. All patients with a strong epidemiological link to malaria should be tested with both rapid antigen detection tests and peripheral blood smears when they are suspected to have malaria.

Our study has several limitations that should be considered. The first is that the retrospective nature of the study makes it prone to information bias from the potentially missing data in the patient's medical records. The second is that the study was conducted in a single center, especially since some malaria cases in the Eastern Province of Saudi Arabia are treated in the primary health care centers, the university and military hospitals, and the private sector. Lastly, the Eastern Province is not an endemic malaria area in Saudi Arabia. All the cases were imported from the endemic Saudi southern areas or from outside the country. Thus, it would be more informative if a similar study was conducted in Tohama coastal area, Jazan region, and the Saudi Arabia-Yemen border to study the complete effects of the COVID-19 pandemic in Saudi Arabia.

## Conclusions

Malaria cases were almost doubled during the COVID-19 pandemic as compared to the pre-pandemic era, signifying the negative effects of the pandemic on malaria epidemiology. The cases increased for a variety of causes that include alternation of health-seeking behaviors, changes in healthcare structures and regulations, and the interruption of malaria preventive services. Future research is needed to study the long-term effects of the changes imposed by the COVID-19 pandemic and to mitigate the effects of any future pandemic on malaria control.

As two patients from our cohort were diagnosed with malaria based on blood smears, although they had negative rapid detection tests (RDTs), we recommend testing all the patients who are suspected to have malaria with both RDTs and peripheral blood smears.
